# Multimodal stimulus coding by a gustatory sensory neuron in *Drosophila* larvae

**DOI:** 10.1038/ncomms10687

**Published:** 2016-02-11

**Authors:** Lena van Giesen, Luis Hernandez-Nunez, Sophie Delasoie-Baranek, Martino Colombo, Philippe Renaud, Rémy Bruggmann, Richard Benton, Aravinthan D. T. Samuel, Simon G. Sprecher

**Affiliations:** 1 Department of Biology, Institute of Zoology, University of Fribourg, Chemin du Musee 10, Fribourg CH-1700, Switzerland; 2 Center for Brain Science and Department of Physics, Harvard University, Cambridge, Massachusetts 02138, USA; 3 Microsystems Laboratory, Ecole Polytechnique Fédérale de Lausanne (EPFL), Lausanne CH-1015, Switzerland; 4 Faculty of Biology and Medicine, Center for Integrative Genomics, University of Lausanne, Genopode Building, Lausanne CH-1015, Switzerland; 5 Interfaculty Bioinformatics Unit and Swiss Institute of Bioinformatics, University of Berne, Berne 3012, Switzerland

## Abstract

Accurate perception of taste information is crucial for animal survival. In adult *Drosophila*, gustatory receptor neurons (GRNs) perceive chemical stimuli of one specific gustatory modality associated with a stereotyped behavioural response, such as aversion or attraction. We show that GRNs of *Drosophila* larvae employ a surprisingly different mode of gustatory information coding. Using a novel method for calcium imaging in the larval gustatory system, we identify a multimodal GRN that responds to chemicals of different taste modalities with opposing valence, such as sweet sucrose and bitter denatonium, reliant on different sensory receptors. This multimodal neuron is essential for bitter compound avoidance, and its artificial activation is sufficient to mediate aversion. However, the neuron is also essential for the integration of taste blends. Our findings support a model for taste coding in larvae, in which distinct receptor proteins mediate different responses within the same, multimodal GRN.

## Abstract (web summary)

While gustatory systems have been extensively studied in adult *Drosophila*, not much is known about taste coding at the larval stage. Here, the authors investigate gustatory receptor neurons in larvae and find single neurons are capable of responding to more than one taste modality.

## Graphical abstract

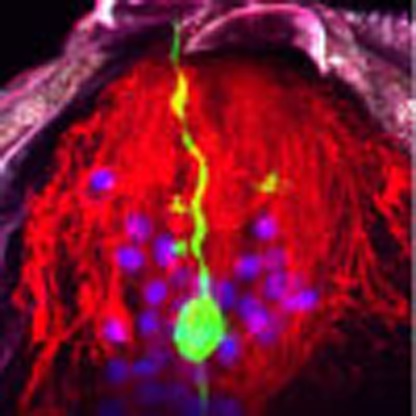

Specialized gustatory receptor neurons (GRNs) provide a first assessment of tastants and thereby deliver essential information regarding the nutritional value, chemical composition and putative toxic compounds of food substrates. Current research supports a labelled line model for taste coding in mammals and in the adult fruit fly, in which each peripheral gustatory cell is tuned to perceive cues of one specific modality such as sweet or bitter. In the mouse, specialized taste-receptor cells (TRCs) are localized in the taste buds on the tongue and the palate epithelium. These TRCs are narrowly tuned and express receptors that are only detecting one of the five basic taste modalities: sweet; bitter; salty; sour; or umami (savoury)^1^.

The taste system of adult *Drosophila* is composed by distinct functional types of GRNs that detect either bitter/high salt, sweet or osmolarity depending on the receptors that they express^1^. Compared with the adult fly the larva has a simple peripheral nervous system with a comparably small number of sensory neurons to assess the properties of its chemical environment. How larvae manage to sample and integrate a wide range of chemicals with few neurons and which of the functional subtypes of GRNs are present remains unknown.

The two main external chemosensory organs are the dorsal organ (DO) and the terminal organ (TO) located at the tip of the head. These contain dendrites of sensory neurons localized in the respective ganglia (DO ganglion (DOG) and TO ganglion (TOG)) (Fig. 1a). Each ganglion contains ∼30 neurons, which have been suggested to function in gustation, olfaction, thermosensation, hygrosensation and mechanosensation^2^,^3^,^4^,^5^. The DOG functions as olfactory organ containing 21 well-described olfactory receptor neurons (ORNs) indicated by the expression of olfactory receptors^6^,^7^. Taste coding is primarily occurring in the TOG. Expression analysis of *Gustatory receptor* genes (*GRs*) in the DOG and TOG identified 10 gustatory sensory neurons (GRNs) by the specific combinatorial expression of 28 *GRs* (ref. 8). Coexpression of *Gr66a* and *Gr33a* in six of these neurons indicates that most larval GRNs might mediate a bitter perception. Other sensory receptor genes in the chemosensory organs include *IRs* (9,10) and receptors of the Pickpocket *(PPK)* family with *ppk11* and *ppk19* playing a substantial role in low salt sensing^11^. However details of gustation coding such as a precise number and molecular function of GRNs remain sparse. Functional studies suggest that external GRNs have an essential role in bitter and salt perception, while internal, pharyngeal GRNs appear to function for sweet-dependent ingestion^11^,^12^,^13^. However, the functional organization of the larval taste system or detailed properties of particular GRNs remain unknown. Here we analyse the properties of individual GRNs in the larva and we show that some sensory neurons are tuned more broadly to different taste modalities, suggesting that the larval taste system is organized different compared with the adult or mammalian system.

## Results

### Larval taste neurons respond to multiple stimuli

**Figure 1:**
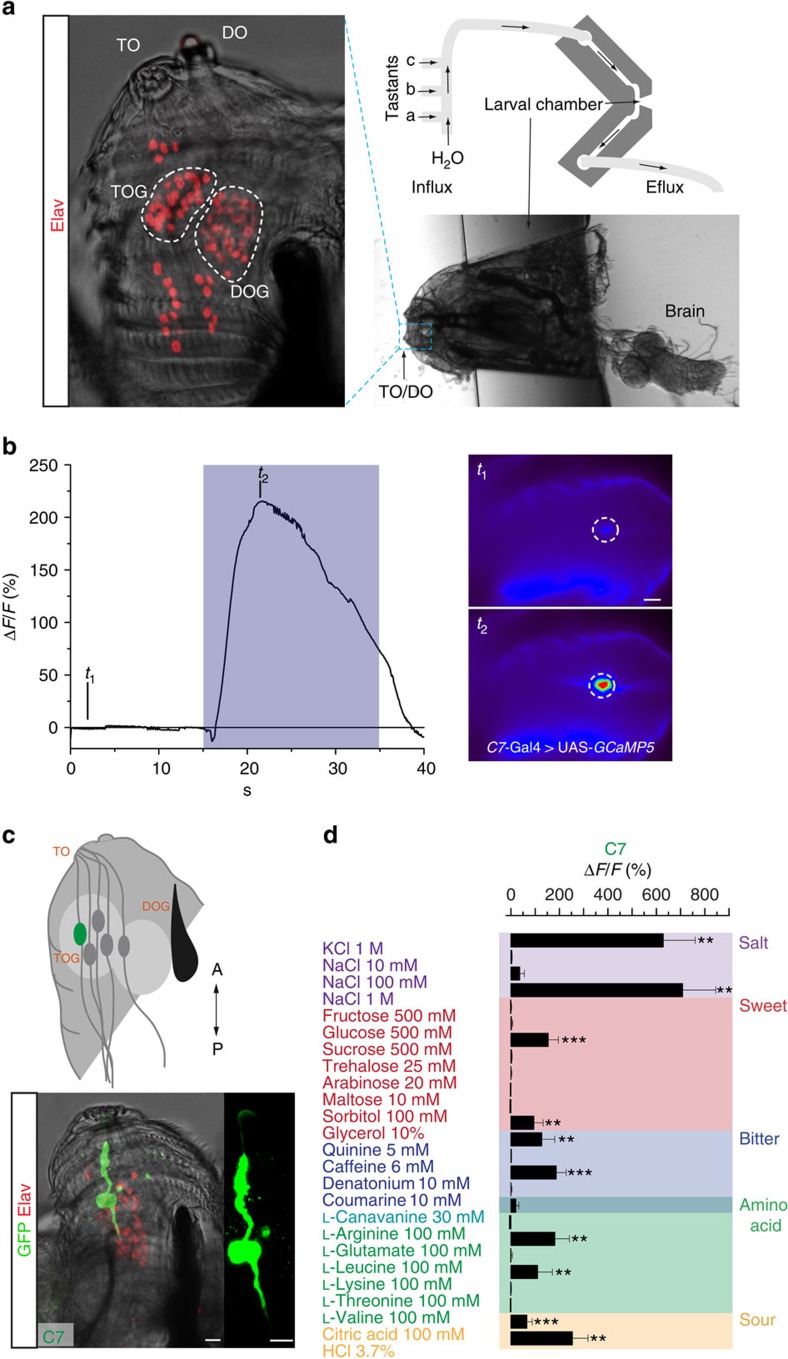
The physiological response of C7 was measured in a specific microfluidic device using genetically encoded calcium sensor *GCaMP5*. (**a**) Elav staining and bright-field image of larval head showing the DO and the TO as well as the related ganglia DOG and TOG. Schema and bright-field image of the microfluidic device. Solutions can be pumped through the influx channels and stimulate the larval chemosensory organs. (**b**) Exemplary measurement of stimulation (NaCl 1 M) of the *GMR57B04*^*ppk6*^ (C7) neuron with *GCaMP5* shows the relative change of fluorescence (Δ*F/F* (%) see materials and methods) for two different time points in false colouration (*t*_1_=beginning of the measurement before stimulation, *t*_2_=maximum of fluorescence change during stimulation). (**c**) Schematic depiction of the selected GRN and staining of GMR57B04^ppk6^-Gal4 crossed with UAS-*mCD8::GFP*. (**d**) C7 shows neuronal responses to multiple stimuli from different taste categories. Changes of fluorescence for a panel of substances measured in the C7 neuron (*n*=5–9). Scale bars, 10 μm. Error bars show the s.e.m. and significances are indicated as following: ***P<0.001 and **P<0.01 in Wilcox signed rank test, samples compared with the baseline when water only was applied.

Electrophysiological recordings of the TO have been proven to be challenging, and the numerous uncharacterized neurons that are housed in these sensilla makes interpretation difficult. To analyse the sensory properties of larval GRNs, we engineered a microfluidic device that enabled us to stimulate sensory neurons in a highly controlled and stereotypic manner, while imaging in a semi-intact preparation (Fig. 1a right panel and Fig. 1b, Supplementary Fig. 5a). This device allows repeated stimulation and application of multiple stimuli for the same animal, thereby facilitating detailed analysis of neuronal responses. We assessed response profiles of individual GRNs by recording neuronal activity using the genetically encoded calcium sensor UAS-*GCaMP5* (ref. 14). Labelling and recording of individual sensory neurons provides us with a tool to access physiological properties of GRNs. Lack of a Gal4 driver that labels exclusively all GRNs makes it reasonable to start analysis of the larval taste system based on function of individual neurons. To identify additional single GRNs, we screened the FlyLight collection^15^ for Gal4 lines showing sparse expression patterns in the sub-oesophageal zone, the primary taste centre in the larval brain^4^,^16^. Stainings of the peripheral tissues of the larva were performed to examine expression in peripheral sensory organs (Fig. 1c; Supplementary Fig. 3c,e, full stack in Supplementary Movie 1). We identified a line (GMR57B04) labelling a previously uncharacterized, individual sensory neuron in the TOG. We named this newly identified neuron C7 according to previous nomenclature^8^ (Fig. 1c).

We tested a selection of chemical cues, which previously were shown to play an important role in taste discrimination for the adult fly^17^,^18^,^19^,^20^,^21^. These chemicals fall into categories that according to the human taste sensation have been classified into salty, sweet, bitter, umami (savoury) and sour. Neuronal activity was recorded for high salt, bitter chemicals like denatonium and quinine, sucrose and glycerol, as well as some amino acids and pH. Unexpectedly, the C7 neuron responded to tastants from all different taste modalities in a highly specific, stereotypic and consistent manner (Fig. 1d). To exclude unspecific responses because of pH or osmolarity, we determined both values for all solutions. By testing substances (glucose, arabinose, sucrose and NaCl) with the same osmolarity we determined that responses for substances with concentrations under 1,000 mOsm kg^−1^ are specific (Supplementary Fig. 5a,b, Supplementary Table 2), while at higher concentrations a response due to osmotic pressure may not be excluded. By testing the same substance (sucrose) with different pH (pH 4, 6 and 9) we find that the response of C7 remains stable, therefore excluding pH-dependent effects.

### Aversive response upon artificial activation of C7

**Figure 2:**
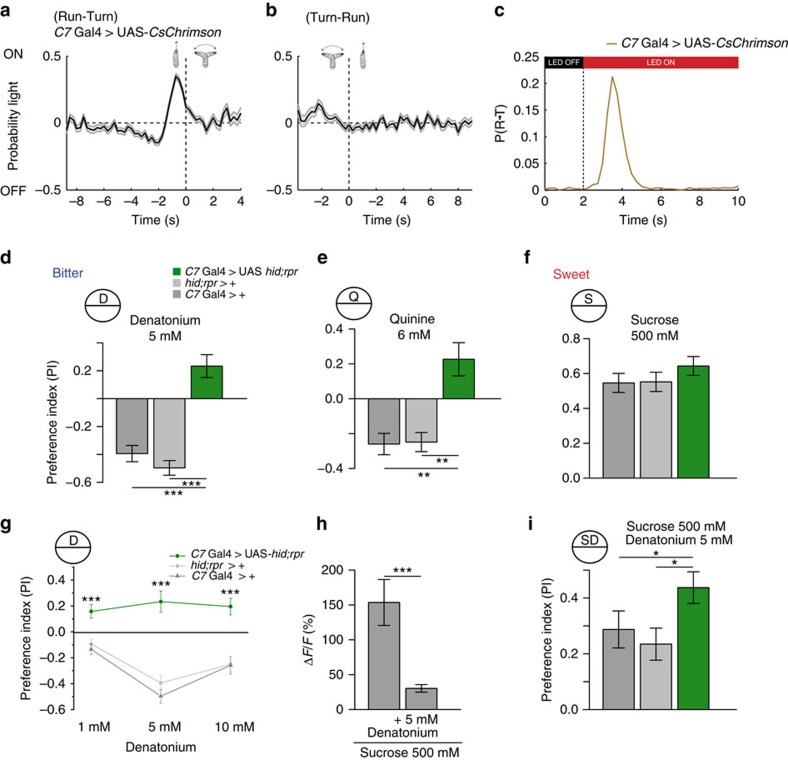
C7 is required for bitter avoidance and discrimination of a sucrose/denatonium mixture. The *C7*-Gal4>UAS-*CsChrimson* larvae show an aversive phenotype after artificial activation. To establish whether navigation decisions are mediated by C7 activity we stimulated *C7*-Gal4>UAS-*CsChrimson* animals with a random flickering red light stimulus. (**a**) On average an increase in optogenetic stimulation is observed before the termination of a run and the initiation of a reorientation (Run-to-turn transition). (**b**) During reorientations of *C7*-Gal4>UAS-*CsChrimson*, increased optogenetic stimulation is present and the reorientation finishes (and a new run is initiated=turn-to-run-transition) when the optogenetic stimulus returns to baseline. (**c**) Stimulating *C7*-Gal4>UAS-*CsChrimson* larvae with a red light step generated an increase in the likelihood of finishing a run and initiating a turn, consistent with avoidance behaviour. (**d**) When expressing *hid;rpr* in the C7 neuron, larvae do not longer avoid denatonium (**e**) or quinine (**f**) while sucrose attraction is not affected (*n*=15) (*C7*-Gal4>+ and *hid;rpr*>+ *P*=0.852 (NS); *C7*-Gal4>+ and *C7*-Gal4>*hid;rpr P*=0.339 (NS); *hid;rpr*>+ and *C7*-Gal4>*hid;rpr P*=0.213 (NS)). (**g**) This phenotype is independent of concentration for 1, 5 and 10 mM denatonium. (**h**) The calcium response in C7 to 500 mM sucrose is inhibited by the concomitant application of 5 mM denatonium (*n*=6–9) and (**i**) C7 is necessary for the reduced preference towards a blend of sucrose and denatonium (*n*=15). Error bars show the s.e.m. and significances are indicated as following: ***P<0.001, **P<0.01, *P<0.05 in Wilcox rank sum test for individual groups and Wilcox signed rank test between two groups.

Since C7 responds to tastants of different valence we wondered whether the artificial activation of this neuron is sufficient to trigger an aversive or attractive response^5^,^22^. To address this question we used an optogenetic reverse-correlation assay, which allows us to assess if activation of C7 mediates navigational behavioural transitions^23^. Larval navigation makes use of two distinct behavioural states: runs, during which the animals uses peristalsis to move forward; and turns, during which the animal sweeps its head to sample the environment and to reorient itself. Depending on the sensory experience larvae modulate the probability to switch between runs and turns. When animals sense an attractive cue during runs, they decrease the probability to start a turn. Conversely if the animal perceives a negative stimulus they are more likely to initiate a turn. Similarly if the larva experiences an attractive cue during a turn, it is more likely to initiate a run, while it will decrease the probability to switch from turn to run if an aversive neuron is activated. We stimulated animals expressing the red-shifted channelrhodopsin *CsChrimson* (ref. 22) in the C7 neuron (*C7*-Gal4/UAS-*CsChrimson*). Animals were exposed to a random flickering of red-light and navigation was simultaneously recorded to determine whether navigational decisions depend on C7 activity^5^. We found that before a reorientation movement (run to turn transition) was initiated, on an average an increase in the optogenetic activation of C7 was observed (Fig. 2a). During reorientation (turn to run transition) events C7 receives decreased optogenetic activation (Fig. 2b). Both these trends are consistent with an aversive response. To confirm this result, we subjected *C7*>*CsChrimson* larvae to a step increase of optogenetic stimulation (light OFF to light ON) and calculated the probability of initiating a reorientation. After an optogenetic activation step larvae showed a strong increase in reorientation probability, consistent with aversive behaviour (Fig. 2c). Therefore artificial activation of C7 suggests, that this neuron can provide information about an aversive cue for directed navigation. Since the C7-Gal4 driver shows in some cases expression in a single ventral pharyngeal organ (VPS) neuron a contribution of this cell to artificial activation experiments using *CsChrimson* cannot be excluded.

### C7 is involved in sensing denatonium/denatonium-containing mixtures

A surprising feature of C7 is that it responded to stimuli that elicit opposing behaviours in the larvae, such as attraction for sucrose and aversion to denatonium or quinine. To test whether the physiological response of C7 to these chemical cues has an impact on the larval behaviour, we performed simple behavioural two choice assays, ablating C7 by specific expression of the pro-apoptotic genes *hid* and *reaper* (ref. 24). As expected, control larvae showed an aversive behaviour towards denatonium (Fig. 2d) and quinine (Fig. 2e), while they were attracted to sucrose (Fig. 2f). Interestingly, animals lacking the C7 neuron did no longer show an aversive response towards denatonium or quinine, but rather displayed a weak attraction towards these chemicals (Fig. 2d,e). This observation is consistent with the fact, that in the reverse-correlation assay artificial activation of C7 elicits a highly aversive behavioural response compared with other GRNs (Supplementary Fig. 1a). Sucrose attraction was not changed by the absence of C7 (Fig. 2f) (*C7*-Gal4>+/*hid;rpr*>+ *P*=0.852 (NS); *C7*-Gal4>+/*C7*-Gal4>*hid;rpr P*=0.339 (NS); *hid;rpr*>+/*C7*-Gal4>*hid;rpr P*=0.213 (NS)). The change from an aversion to an attraction towards bitter chemicals cannot be correlated to osmolarity, as different concentrations of denatonium displayed the same level of attraction (Fig. 2g).

Since C7 is necessary for aversive behaviour to bitter stimuli but additionally reacts to sweet cues, we wondered whether it might function in the integration of information about tastants with different valence. We therefore tested if the exposure to a blend of sweet and bitter substances elicits different responses as compared with the pure substances. First, we monitored the *GCaMP5* response to sucrose supplemented with 5 mM denatonium in the C7 neuron. Intriguingly denatonium showed a strong inhibition of the sucrose response (Fig. 2h). Thus, even though C7 responded strongly to sucrose and denatonium when presented alone, the sensory responses were not additive but rather cross-inhibitory.

We therefore next tested whether the genetic ablation of C7 affected sucrose preference when supplementing with denatonium (Fig. 2f). Control animals showed a lower attraction to sucrose containing 5 mM denatonium. Animals lacking C7 displayed a stronger preference for the blend than control animals (Fig. 2i). Even though C7 has no impact on the preference when sucrose is presented alone, the discrimination of a sucrose denatonium blend requires functional physiological repression of sucrose by denatonium in this neuron, as animals lacking C7 fail to appropriately evaluate this blend. Overall these results show, that C7 is required to maintain an appropriate perception of pure substances or substance mixtures with different valence. The lack of C7 results in an elevated attraction towards tested chemicals.

### IR25a is involved in denatonium sensing

**Figure 3:**
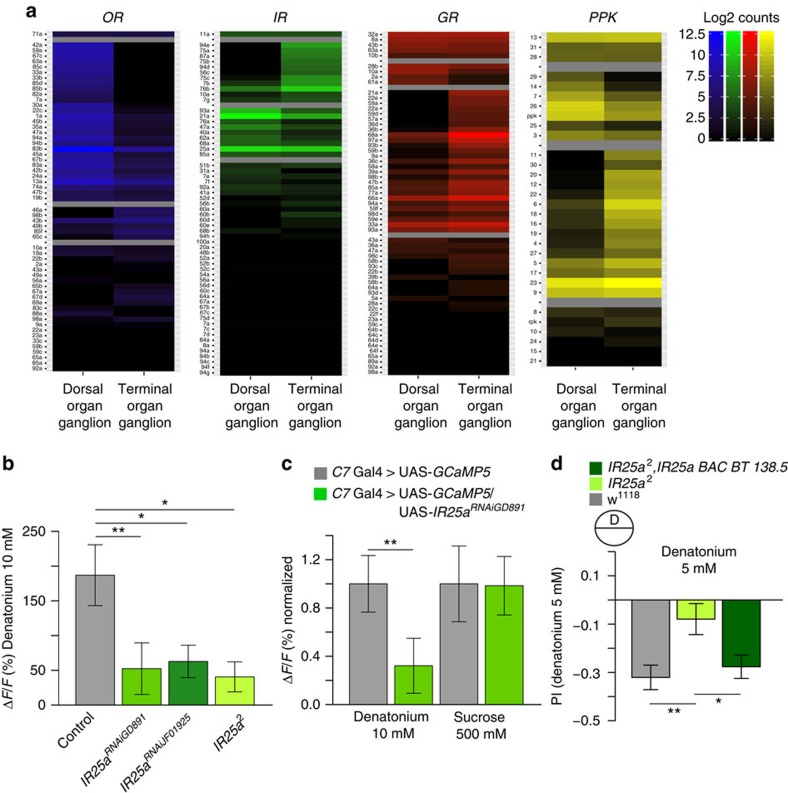
*IR25a* mediates the response to denatonium but not to sucrose in C7. (**a**) Illumina sequencing of RNA extracted by either the DOG or the TOG show regulation of olfactory receptors, IRs, GRs and PPKs. Heatmaps show the log2-transformed mean expression values. The genes in each heat map are classified according to their expression level into four classes and are separated by grey horizontal lines. The classes from top are: expression in both organs with log2-fold change (FC<1.5), higher or only expressed in DOG (FC>1.5), higher or only expressed in TOG (FC>1.5), low or no expression in either organ (read count<10). Detailed expression data is included in Supplementary Data 1. (**b**) Expression of *IR25a*^RNAi^ in the C7 neuron reduces the ΔF/F (%) to denatonium (10 mM) significantly (*IR25a*^RNAiGD891^: *P*=0.004 and *IR25a*^RNAiJF01925^: *P*=0.03) with two different RNAi Lines or in the *IR25a*^*2*^ mutant background (**c**) but has no influence on the sucrose response (*P*=0.797). (**d**) The *IR25a*^*2*^ mutant shows significant problems to discriminate between neutral agarose and denatonium this phenotype can be restored by a rescue construct BAC138.5 (*n*=15–17). Error bars show the s.e.m. and significances are indicated as following: **P<0.01 and *P<0.05 in Wilcox rank sum test for individual groups and Wilcox signed rank test between two groups.

Since C7 is involved in mediating different behaviours, this neuron might serve the animal as a broadly tuned sensor, integrating taste information and setting context dependent valence already at the sensory level. Different families of chemoreceptors have been shown to be involved in sensing tastants including *GRs*, *IRs* and *PPKs* (ref. 11). To understand the molecular mechanisms underlying such a coding, we performed transcriptomic analysis using RNAseq on the two main chemosensory organs to yield a set of candidate receptor genes for gustatory cue detection (Fig. 3a; Supplementary Data 1). We tested highly expressed receptor genes (*Ir25a*, *Gr68a*, *Gr47b*, *Gr36b* and *ppk6*) specifically in C7 by cell-type specific transgenic RNAi while analysing calcium responses to denatonium (Supplementary Fig. 2). We found that C7 showed a significant decreased denatonium response when knocking down *IR25a* using two independent RNAi lines (Fig. 3b), while RNAi against *Gr68a*, *Gr47b, Gr36b* or *ppk6* did not alter the response (Supplementary Fig. 2b). This result was confirmed by recording the calcium response to denatonium in C7 in the *IR25a*^*2*^ mutant background. Interestingly RNAi *IR25a* had no impact on the sucrose response (*P*=0.797) (Fig. 3c). In C7, *Ir25a* seems to have a specific role in the detection of denatonium, since in *Ir25a*^*RNAi*^ the physiological sucrose response remained unaffected (Fig. 3c). We next tested *IR25a*^*2*^ mutant larvae in denatonium avoidance behaviour. We indeed found that *IR25a*^*2*^ mutants showed decreased denatonium avoidance. This defect in denatonium avoidance can be restored by introducing expression of *IR25a* with a BAC rescue construct 138.5 (Fig. 3d). These results suggest that different tastants within the same neuron might use different receptor molecules and or signal transduction pathways as appearing in *Caenorhabditis elegans*^25^. In addition, we tested the role of *IR25a* in the denatonium-mediated inhibition of the sucrose response in C7. Intriguingly, this process seems to be mediated by *IR25a*, as mutant animals fail to discriminate a sucrose denatonium mixture and show a preference similar to sucrose only (Supplementary Fig. 6a). The physiological inhibition in C7 of the sucrose response by denatonium is not occurring in animals with a cell-specific knockdown of *IR25a* using RNAi (Supplementary Fig. 6b). However, the detailed molecular mechanisms of this interaction remain elusive. In the adult olfactory system, *IR25a* functions as co-receptor for other *IRs* (ref. 26). Similarly, in C7 *IR25a* seems to be a part of a receptor complex, as the calcium response to denatonium is not fully abolished in the mutant background.

**Figure 4:**
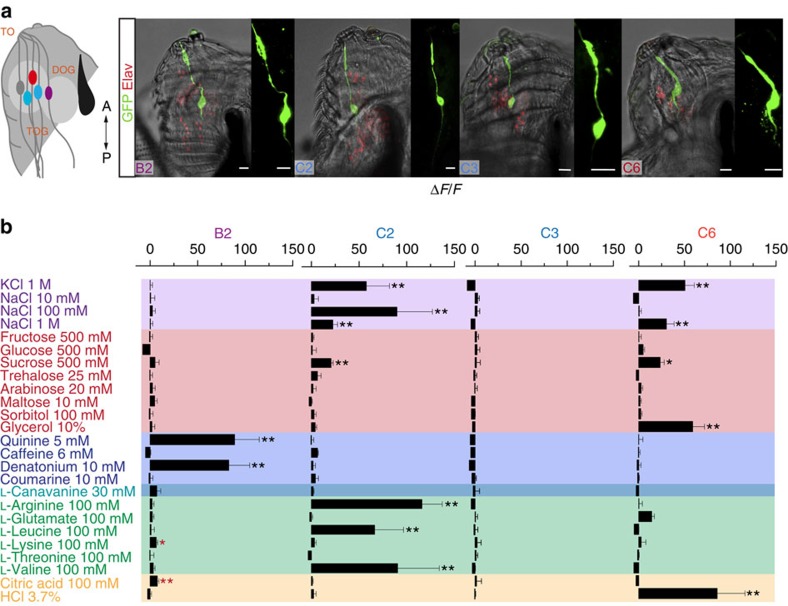
Larval GRNs show different degrees of multimodality. (**a**) Immunostaining of *B2*-Gal4, *C2*-Gal4, *C3*-Gal4 and *C6*-Gal4 crossed with UAS-*mCD8::GFP*. Scale bars, 10 μm. (**b**) Neuronal response profiles of the four GRNs tested for different tastants. The neurons show different response profiles with some tuned very narrowly (B2) and others multimodal (C2 and C6). Error bars show the s.e.m. and significances are indicated as following: **P<0.01 and *P<0.05 in Wilcox signed rank test, samples compared with the baseline measurements with water only. (Red asterisks mark d*f*/*f* changes under 10%.)

To test if multimodality is a general principle of the larval gustatory system, we examined the response profiles of other recently described GRNs: C2, C3, the CO_2_ sensitive C6 (ref. 27) and B2, which has its cell body in the DOG but receives information from the TO (ref. 8) (Fig. 4a, Supplementary Fig. 4). These GRNs had different types of responses: the B2 neuron exhibited a classical narrowly tuned response profile towards bitter chemicals; C2 and C6 were more broadly tuned, similar to the C7 neuron. C3 did not respond to any chemical in our panel (Fig. 4b). Since C6 shows particularly strong responses when exposed to tastants at high concentrations it appears possible that C6 might sense osmotic pressure. Interestingly even though B2 shows a physiological response to denatonium, when tested in a two choice assay, animals lacking B2 performed indistinguishable from the genetic controls (Supplementary Fig. 6c). This experiment indicates, that different GRNs might play a role in different behaviours, as C7 is necessary for denatonium avoidance in a two choice situation. The larval taste system seems, therefore, to be highly complex and different sets of GRNs must be involved in the detection of different mixtures of gustatory chemicals.

## Discussion

In the adult fly, taste perception is mediated by specialized GRNs that are narrowly tuned to specific taste modalities: sweet; bitter; water; and osmolarity. Different subtypes of bitter- or sweet-sensitive GRNs exist, which mediate perception of different sweet or bitter substances^28^,^29^,^30^. Recent findings suggest a complex and integrative mechanism of taste perception in the fruit fly, already at the sensory level^11^,^31^,^32^,^33^,^34^.

Since in mouse and adult flies TRCs appear to strictly transduce information about one specific stimulus class, it is assumed that this provides a general principle of taste coding. The existence of multimodal GRNs in the *Drosophila* larvae raises two questions: first, is this a more widely used principle of taste coding; and second, under which conditions is multimodal gustatory coding beneficial.

The *Drosophila* larva contains a comparably simple nervous system with an astonishing repertoire of behaviours related to chemical signals that span from simple repulsive/attractive responses to complex computation including learning and the formation of memory^35^,^36^. Our findings support gustatory coding with one neuron covering the perception of different tastants of unequal valence. Bitter substances are perceived by C7 and the neuron has a major impact on the aversive response towards quinine and denatonium. This result shows, that the weight of a substance is defined at the sensory level. Animals lacking C7 can still sense these chemicals but are now instead attracted to them, indicating that other GRNs are also involved in sensing these stimuli. Compared with unimodular sensory neurons, the C7 neuron responds additionally to sweet tastants. We showed that denatonium is suppressing the sucrose answer and that this suppression is necessary for the appropriate preference towards a sucrose denatonium blend. These results suggest, that this multimodal neuron plays different roles depending on the combination of tastans. This could enable *Drosophila* larvae to perceive a complex environment with a small number of GRNs.

A similar type of coding has been described in *C. elegans*, which also has a small number of sensory neurons^25^. However, in this case, multimodal neurons mediate directed behaviours, either attraction or repulsion: these neurons respond to different signals of the same valence of different sensory modalities, including temperature and chemical compounds. Here, transduction of different noxious stimuli has been shown to involve different signal molecules. For example *OSM-10* (a cytosolic protein) is involved in avoiding hyperosmolarity, *qui-1* (a protein with WD-40 repeats) was connected for quinine avoidance but not hyperosmolarity. Thus artificial activation for example of the ASH neuron elicits a repulsion behaviour^25^.

Similarly, artificial activation of the multimodal gustatory C7 neuron results in a strong aversion behaviour, compared with other multi- or unimodal GRNs. However in contrast to the ASH neuron of *C. elegans*^25^, C7 is activated by chemical cues of different valence. Our data suggest that depending on the exact composition of chemicals in the environment, different subsets of neurons might be recruited. Therefore, the impact of C7 on behaviour depends on the population of sensory neurons that is activated but is restricted to a negative influence of the perception of individual substances or blends. Calcium imaging recordings are not sufficient to explore properties of the neuronal response fully. Compared with electrophysiological recordings, it is not possible to extract detailed properties such as depolarizing or hyperpolarizing changes of the membrane potential or the action potential frequency. If modulations through gap junctions or other cell–cell interactions between GRNs exist in the larvae or if chemicals interact at the receptor level, like shown for the adult chemosensory system remains to be explored^34^,^37^. The specific role of *IR25a* in denatonium sensing suggests that the larva enlarges the ability to sense a broad spectrum of tastants in two steps: by using different receptor molecules for individual responses in the same neuron and through a combinatorial activation of different subsets of GRNs. It will be highly interesting to analyse further receptor candidates involved in the mechanisms discovered here. Our receptor list provides a list of candidates and a more extensive screen will be necessary to determine the full complement of receptor subunits for denatonium and sucrose detection. In this paper we show the existence of a new type of GRN. However, further studies will be necessary in order to dissect the detailed molecular properties of multimodal taste coding.

## Methods

### Immunofluorescent stainings

The mouthparts of the third instar larvae were dissected in phosphate-buffered saline (PBS) and subsequently fixed in a solution of PBS with paraformaldehyde (4%) for 20 min. The preparations were washed several times with PBT every 45 min and then incubated with the following primary antibodies over night at 4 °C: rat anti-Elav, mouse anti-β-Gal (Developmental Studies Hybridoma Bank; dilution 1:20) and rabbit anti-GFP (Molecular probes; dilution 1:1,000). After being washed every 45 min for at least 6 h in PBT the preparations were incubated with secondary antibodies again over night at 4 °C: anti rat Alexa-647, anti mouse Alexa-568 or anti rabbit Alexa-488 (Jackson Immunoresearch). All secondary antibodies were developed in goat or donkey and applied at dilution 1:200. After this step, the preparations were washed twice 15 min in PBT and twice 15 min in PBS, mounted in 50% Glycerol (Sigma Aldrich, St Louis) and imaged with a TCS Leica SP5 confocal microscope and later processed with LASAF and arranged in Adobe Photoshop.

### Tastants

For calcium imaging recordings, tastants were dissolved in Millipore water. All tastants were stored as recommended and dissolved aliquots were stored in 4 °C for no more than 2 weeks. All tastants were obtained from Sigma-Aldrich (St Louis, MO). Tastants were tested in the following concentrations, unless otherwise indicated: D-(−)-arabinose (ARA), 20 mM; L-arginine (ARG), 100 mM; caffeine (CAF), 5 mM; L-canavanine (l-Cana), 30 mM; citric acid (CIA), 100 mM, pH 2; coumarine (COU), 10 mM; denatonium benzoate (DEBE), 5 mM, 10 mM; D-(−)-fructose (FRU), 500 mM; D-(+)-glucose (GLU), 500 mM; L-glutamic acid monosodium salt hydrate (GLT), 100 mM; glycerol (GLY), 10%; HCl (HCl), 3.7%, pH 2.5; L-leucine (LEU), 100 mM; L-lysine monohydrochloride (LYS), 100 mM; D-(+)-maltose monohydrate (MAL), 10 mM; sodium chloride (NaCl), 10 mM, 100 mM, 1 M; D-sorbitol (SOR), 100 mM; D-(+)-sucrose (SUC), 500 mM; quinine hemisulfate salt monohydrate (QUI), 6 mM; L-threonine (THR), 100 mM; D-(+)-trehalose dehydrate (TRE), 25 mM; L-valine (VAL), 100 mM.

### Calcium imaging

For calcium imaging recordings early stage third instar larvae were dissected in modified AHL-saline (NaCl, 108 mM; KCl, 5 mM; MgCl2, 8.2 mM; NaHCO3, 4 mM; NaH2PO4, 1 mM; HEPES, 5 mM; pH 7.5, in Millipore water). The tip of the head was introduced into the chamber, assuring that the chemosensory organs were exposed to the liquid passing through the channel. To ensure that the channel was closed and the brain persisted in an adequate osmolarity, a drop of 2% agarose diluted in AHL saline was introduced on top of the brain. Measurements were carried out as followed: 100 frames (F) (85 ms F^−1^) period of washing substance (Millipore water) followed by a 200 F period of stimulation and again a period of at least 100 F with the washing substance.

For the stimulation of the chemosensory organs, liquids were pumped by mp6-micropumps (Bartels Mikrotechnik GmbH, Dortmund) via a 0.3 mm tubing (VWR) through the Microfluidic chamber with an average flow rate of 7,7 ml min^−1^.

Changes in fluorescence were calculated as followed:


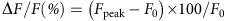


*F*_0_ was calculated from five frames during the unstimulated phase of the first 100 frames time period. *F*_peak_ was taken as the point of highest intensity during the measurement (time during stimulation). For the analysis of calcium imaging measurements we used the LASAF Software (Leica) and calculated fluorescence changes in Microsoft Excel 14.4.5. Individual animals were stimulated with randomly chosen combinations of tastants. Repetition of combinations was avoided and individual animals were stimulated with a maximum of four tastants. High osmolarity and low or high pH were only tested at the end or alone to avoid tissue damage or desensitization. Data in Fig. 2h were acquired from animals stimulated individually with sucrose, denatonium or a mixture of both chemicals in order to avoid cross-reaction or desensitization.

### Chip microfabrication

For the micro fabrication of the master moulds for the chip a 2 μm thick layer of SU8-1040, followed by a 300 μm thick layer of SU8-1075 were spined and on a silicon wafer and patterned by photolithography. To produce the microfluidic chips for gustatory stimulation of the larvae, a 10:1 polydimethylsiloxane (PDMS) mixture (Dow Corning) was purred on the mould and cured for at least 1 h at 80 °C. The chips were cut and holes were punched for the connection with the tubing system. Afterwards, the chips were bonded by plasma oxygen (0.6 mbar, 100 W for 0.1 min) to a glass cover slide.

### Fly genetics

For the experiments the following strains were used: kindly provided by J. Carlson: *Gr97a*-GAL4; *Gr94*-GAL4; and *Gr10a*-Gal4. *Gr21a*-GAL4 and *GMR57B04*-GAL4 (Bloomington); UAS-*GCamP5* (L. Looger); w1118; wtCS; UAS-*mcd8 GFP*; UAS*-hid;rpr*; *UAS-nLacZ*; *UAS-mcD8 GFP*; *IR25a*^*2*^(ref. 38); and *IR25a*^*2*^*;IR25a-*BAC (ref. 39). UAS-*CsChrimson* flies were a gift from Vivek Jayaraman.

### CsChrimson behaviour experiments

Eggs were collected from grape juice plates. Larvae were reared in the dark and fed with 0.5 mM ATR. Based on the spiracle development, we selected late second instar larvae. Groups of 30 larvae were placed in the central region of 22 × 22 cm 2% agar plate at the beginning of each experiment.

To illuminate the behavioural arena we used infrared LED bars (875 nm), and for optogenetic stimulation we used a panel of 625 nm LEDs with a controlled uniform intensity in the behavioural arena of 1.9 W m^−2^. Animals were recorded using a CCD Mightex camera containing a long-pass infrared filter (740 nm) at 4 Hz. Random flickering stimuli was synchronized with image acquisition. Probabilities for Fig. 2a–c were computed as the number of animals initiating a turn in a 0.25 s time bin divided by the total amount of animals in the behavioural arena. The random flickering stimulus used for Fig. 2a,b was generated using a Bernoulli process, the simplest white process (flat power spectrum). For the calculations of triggered averages we considered the ON state of the red LEDs as +1 and LEDs OFF state as −1. The flickering was synchronized with image acquisition. We identified all the reorientation events using MAGAT analyser (ref. 40) and then extracted the stimulus sequences that preceded the initiation of each reorientation and each run using custom code written in MATLAB. The calculation of the triggered averages was made following (Dayan and Abbott, Theoretical Neuroscience).

### Two choice assay

For the two choice assay, 2.5% of agarose was filled in Petri dishes and one-third of the cooled agarose was removed and immediately refilled with another agarose solution (2.5%) that contained the second respective stimulus. The following chemicals were used: Denatonium (5 mM) or D-(+)-sucrose (500 mM) from Sigma-Aldrich (St Louis, MO). At the beginning of the experiment, 30 five-day old feeding L3 larvae were placed along the midline in a neutral zone (defined as 1 cm zone in the middle of the petri dish). After 5 min the larvae on both sides were counted and the preference index (PI) calculated as followed:


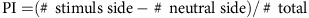


R 3.1.1 was used for data analysis and depicting the behavioural experiments. We applied the Wilcox rank test to verify statistic significances between two data groups. Significance levels are *P*<0.05 (*), *P*<0.01 (**) and *P*<0.001 (***).

### RNAseq of sensory organs

For RNAseq experiments the protocol for the preparation of a cell suspension after Egger *et al*.^41^ was slightly modified and applied as followed.

For dissection, 5-day old feeding L3 larvae were collected and washed in 70% ethanol followed by PBS (two repetitions). Forty to fifty mouthparts (MPs) were dissected in Schneider's Medium (Gibco), than rinsed in fresh medium and subsequently transferred to a siliconized microcentrifuge tube with 1-ml Rinaldini solution. From here on, experiments were performed under laminar flow in sterile conditions. The MPs were centrifuged for 6 min at 900*g* at RT. The supernatant was carefully removed and MPs were washed in Rinaldini solution. The Rinaldini solution was replaced by 1 ml collagenase I (0.5 mg ml^−1^, sterile filtered, Sigma-Aldrich). The digestion was allowed to take place for 40 min at RT and subsequently, the tissue was centrifuged 6 min at 850*g* at RT and collagenase was replaced by Schneider's medium. After 6 min of centrifugation at 750*g* at RT all the supernatant was removed and 10 μl of Schneider's medium per MP were added. With a Pipette, set to half the contained volume, the solution was pipetted up and down 100–200 times.

The suspension was pipetted on a small petri dish containing Sylgard (Dow Corning) and organs were manually sorted under a fluorescent microscope with a glass capillary (GC100TF-10, Harvard Appartus) and subsequently transferred in 100 μl of Lysis Buffer (From PicoPure RNA Isolation Kit, Arcturus). The RNA extraction was performed as suggested by the Kit protocol. Samples were additionally treated with DNAse (Quiagen, catalog 79254).

After RNA extraction, samples were treated exactly as recommended in SMARTer Ultra Low RNA Kit for Illumina sequencing (PT5163-1 (051013)) to transcribe RNA into cDNA and purify the samples.

The cDNA volume was reduced from 10 to 1 μl by using speed vac for ∼9 min. Libaries were prepared using the TruSeq DNA Sample Preparation Guide without gelsize selection, quality was checked after covaris shearing by Bioanalyzer 100 and Qubit. Samples were paired-end sequenced (2 × 100b) on an Illumina HiSeq2500 instrument.

The quality of the sequence reads was assessed using fastQC version 0.10.1 (http://www.bioinformatics.babraham.ac.uk/projects/fastqc/). The reads were mapped to the *Drosophila melanogaster* genome assembly (dm3) using the spliced alignment tool TopHat2 (v2.0.6) with default parameters^42^. The number of reads that map to annotated genes (release 5.12) was counted with HTSeq-count (ref. 43). To test for significant differences of gene expression levels between DOG and TOG the R/bioconductor-package DESeq2 (ref. 44) was applied. The *P* values were corrected for multiple testing following^45^ and threshold of 0.05 was applied. The R/bioconductor-package DESeq2 (ref. 44) was used to perform a differential gene expression analysis between DOG and TOG. A size-factor normalization was employed to account for differences in the sequencing depth of the samples. A generalized linear model was fitted to the data, allowing the error distribution to follow a negative binomial function. The significance of the observed differences was determined by means of a Wald test. The *P* values were corrected for multiple testing following^45^ and a threshold of 0.05 was applied. Expression of sensory receptor genes within the TOG and DOG are summarized in Supplementary Data 1.

### Determination of pH and osmolarity

PH of solutions was measured using a pH meter (Mettler Toledo MP225) and osmolarity of solutions was determined by using an Osmometer (Fiske, Model 210 ‘Micro osmometer'). All solutions were measured at room temperature and in the concentrations used for the experiments.

### Statistics

To compare between two groups in the behavioural two choice assays we applied the wilcox signed rank test. To determine significances for calcium responses in individual neurons, we assessed an individual baseline for each neuron, determining the fluorescence chance when water only was applied to correct for bleaching or other biological variability's during the measurements (find H_2_O measurements and statistics for all substances in Supplementary Table 2). Significance levels are *P*<0.05 (*), *P*<0.01 (**) and *P*<0.001 (***).

### CsChrimson behaviour experiments

In Fig. 2a,b the shaded regions correspond to the standard error of the mean. For each curve in Fig. 2a–c and Supplementary Fig. 1 between 115 and 124 animals were used. In Fig. 2c and Supplementary Fig. 1, each data point is a probability and the mean of the distribution of larvae undergoing a run-to-turn transition (larvae undergoing this transition were considered as 1 and larvae that did not undergo this transition as 0). For all the curves the amount of data collected satisfies np>=5 and *n* (1−*p*)>=5 (*n* is the number of samples, and *p* is the probability of undergoing a transition); therefore a *z*-test was conducted to compare the peaks of the response that follows opotgenetic stimulation with the unstimulated run-turn distribution. For B2, C6 and C7 the run-turn distribution at the peaks were significantly higher than the unstimulated run-turn distribution with *P*<0.01.

## Additional information

**Accession codes**: Sequence Read Archive (SRA) ID: SRP065737.

**How to cite this article:** van Giesen, L. *et al*. Multimodal stimulus coding by a gustatory sensory neuron in *Drosophila* larvae. *Nat. Commun.* 7:10687 doi: 10.1038/ncomms10687 (2016).

## Supplementary Material

Supplementary Information Supplementary Figures 1-6 and Supplementary Tables 1-2

Supplementary Data 1 RNA seq information of sensory receptor genes

Supplementary Movie 1 The movie represents a 3D image sequence acquired with a confocal microscope of a C7-Gal4 UAS-mCD8::GFP larval CNS
