# Erratum: Multimodal stimulus coding by a gustatory sensory neuron in *Drosophila* larvae

**DOI:** 10.1038/ncomms11028

**Published:** 2016-03-14

**Authors:** Lena van Giesen, Luis Hernandez-Nunez, Sophie Delasoie-Baranek, Martino Colombo, Philippe Renaud, Rémy Bruggmann, Richard Benton, Aravinthan D. T. Samuel, Simon G. Sprecher

Nature Communications **7**: Article number: 1068710.1038/ncomms10687 (2016); Published: 02112016; Updated: 03142016.

The affiliation details for Martino Colombo, Rémy Bruggmann and Richard Benton are incorrect in this article. The correct affiliations for these authors are listed below. Martino Colombo, Rémy BruggmannInterfaculty Bioinformatics Unit and Swiss Institute of Bioinformatics, University of Berne, Berne 3012, Switzerland. Richard BentonFaculty of Biology and Medicine, Center for Integrative Genomics, University of Lausanne, Genopode Building, Lausanne CH-1015, Switzerland.

