# Supplementary material for: Multimodal stimulus coding by a gustatory sensory neuron in Drosophila larvae
Source: Nat Commun. 2016 Feb 11;7:10687. doi: 10.1038/ncomms10687 (PMC4753250; doi:10.1038/ncomms10687)
Supplement: Supplementary Information — Supplementary Figures 1-6 and Supplementary Tables 1-2 [file ncomms10687-s1.pdf]

a

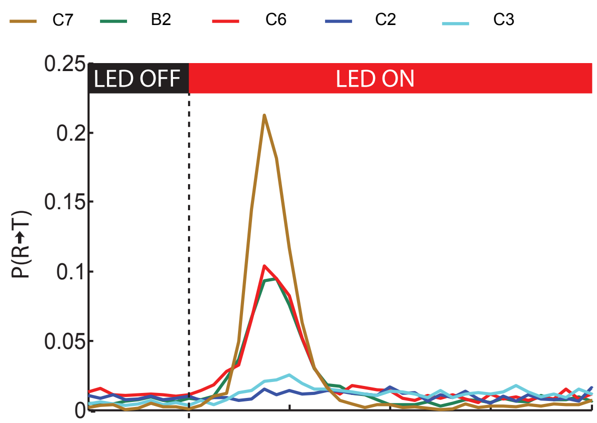

Supplementary Figure 1.: Optogenetic step increase to the five GRNs.

Exposure to an optogenetic step increase leads to an enhanced probability of a run-to-turn transition for *B2*-Gal4>UAS-*CsChrimson*, *C6*-Gal4>UAS-*CsChrimson* and strongly for *C7*-Gal4>UAS-*CsChrimson*. *C2*-Gal4>UAS-*CsChrimson* and *C3*-Gal4> UAS-*CsChrimson* do not show any behavioral change.

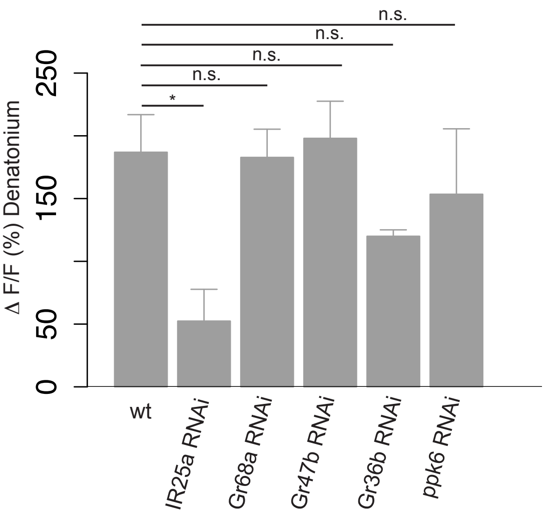

Supplementary Figure 2.: Expression of taste related receptor genes in the two main chemosensory organs.

(**a**) RNAi against the most abundant receptors in the C7 neuron shows, that *IR25a*, but not *Gr68a* (p=1), *Gr47b* (p=0.833), *Gr36b* (p=0.524) or *ppk6* (p=0.435) is responsible for the denatonium response in this neuron (n=5-11). Error bars show SEM. Significances for the calcium measurements are indicated as following: *** p<0.001, ** p<0.01, * p<0.05 in Wilcox signed rank test.

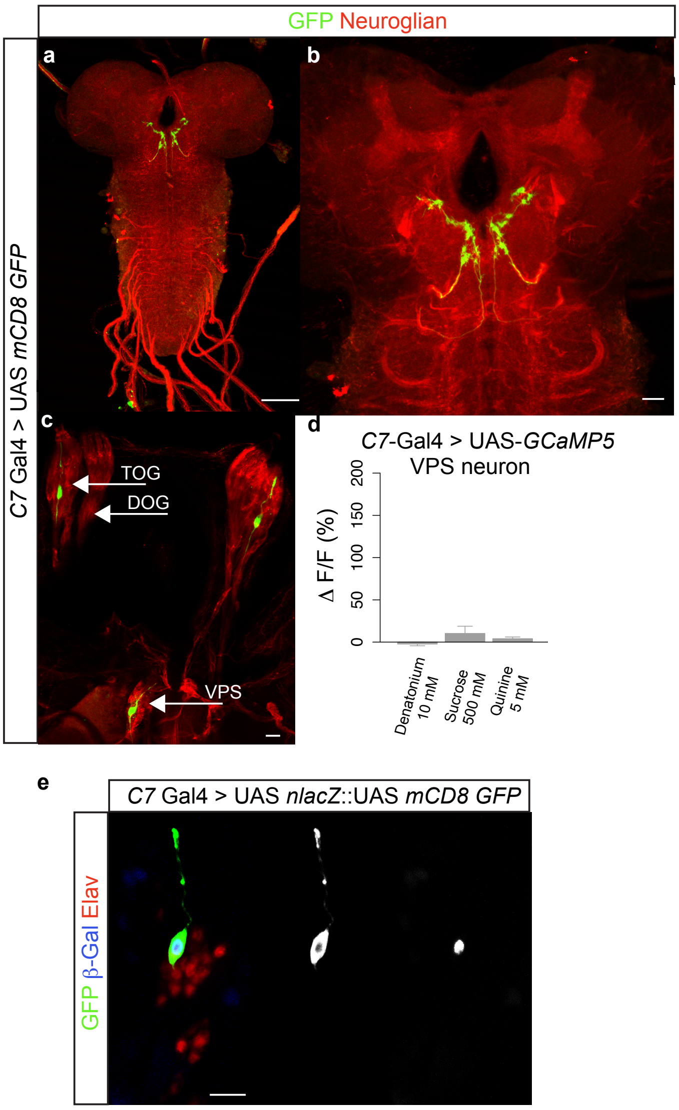

Supplementary Figure 3.: Expression pattern of *GMR57B04*-Gal4

Immunostaining of *C7*-Gal4 > UAS-*mCD8 GFP* animals in the brain **(a)** scale bar 50 µm **(b)** Scale bar 10 µm **(c)** and the periphery. Labeling is observed in one cell in the TOG and in some cases in a pharyngeal neuron located in the DPS. **(d)** Measurements of neuronal activity in the DPS neuron showed no response to denatonium (10 mM) or quinine (5 mM) and only a very low reponse to sucrose (500 mM). (e) Staining of *C7*-Gal4 > UAS-*nlacZ*::UAS-*mCD8 GFP* shows labeling of one cell body and nucleus in the TOG. Scale bars 10 µm.

a

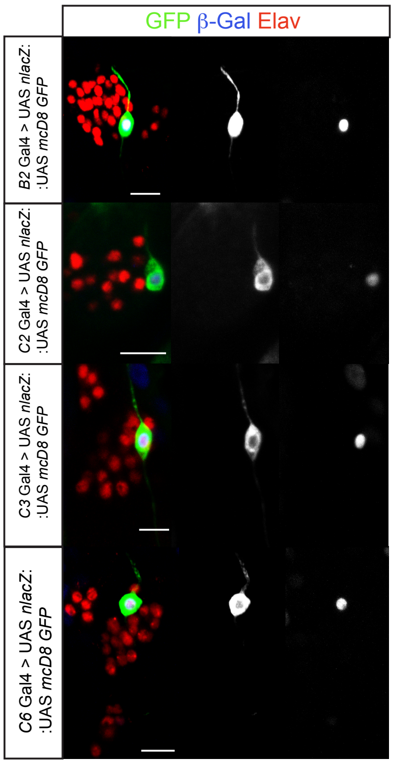

Supplementary Figure 4

Staining of the four different Gal 4 drivers (B2, C2, C3, C6) crossed with UAS-*nlacZ*::UAS *mCD8 GFP* show labeling of individual cell-bodies (GFP) and nuclear staining (ß-Gal). Scale bars 10 µm.

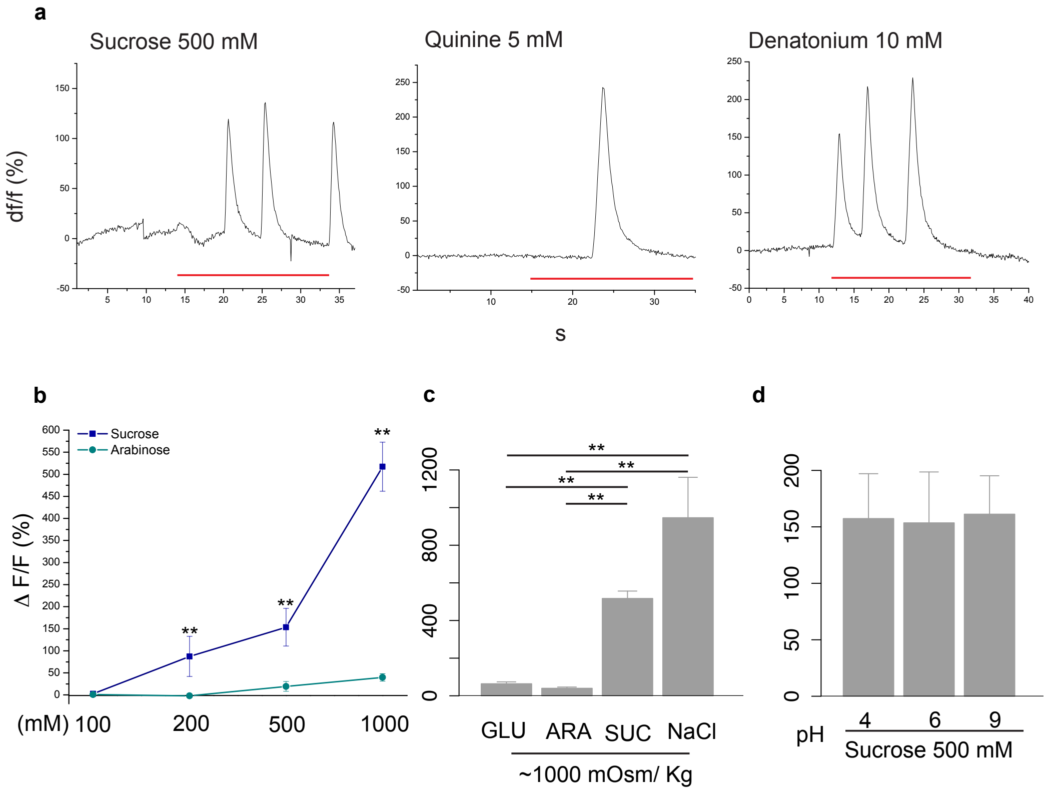

Supplementary Figure 5

(a) Exemplary calcium traces for different responses in the C7 neuron, red bar indicates time of stimulation. **(b)** Sucrose and arabinose were applied in different concentrations and fluorescent change in *C7*-Gal4>UAS-*GCaMP5* animals were measured. Sucrose shows a concentration dependent reaction strength while arabinose only elicits weak changes in concentrations over 500 mM. **(c)** Compared neuronal responses to tastants with approximately the same osmolarity show that responses are lower for non specific substances like glucose (GLU) and arabinose (ARA) and stronger for specific substances sucrose (SUC) and NaCl. **(d)** Changement of the pH of Sucrose doesn't lead to a change in response strength (n=5-9) (pH4/pH6 p=0.797; pH6/pH9 p=0.699; pH4/pH9 p=0.841). Error bars show the SEM. Significances between groups (sucrose and arabinose) are indicated as: ** p<0.01in Wilcox signed rank test.

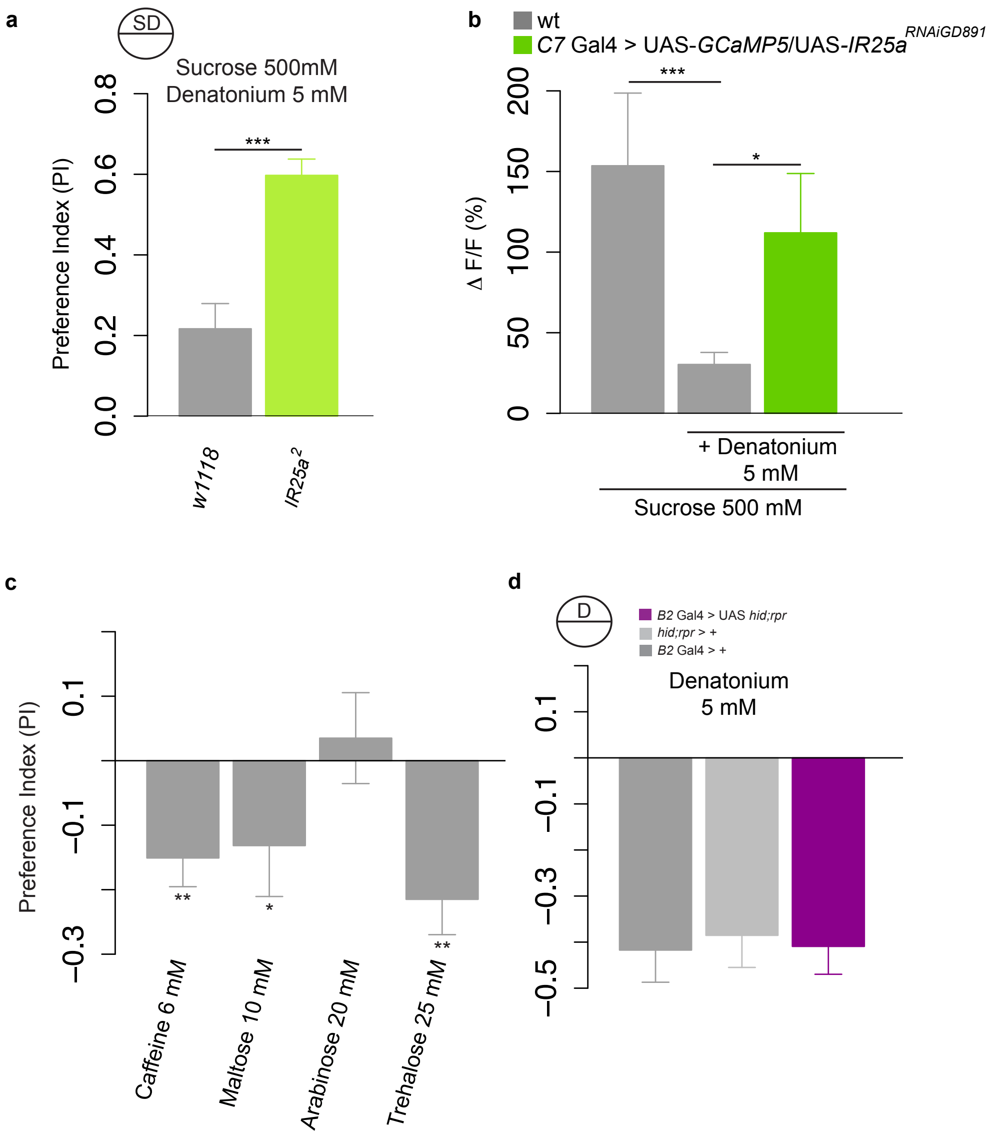

Supplementary Figure 6

(a) *IR25a*^{2} mutant larvae fail to discriminate a mixture of sucrose (500 mM) and denatonium (5 mM), compared to the control animals (n= 15; p= 0.00) in a two choice assay. **(b)** Concurrent with this, animals with knockdown of the *IR25a* receptor specifically in C7 (*C7*-Gal4 > UAS-GCaMP5/UAS-*IR25a*$^{\mathrm{RNAiGD891}}$) do not display the reduced calcium response when stimulated with a solution containing sucrose and denatonium (p=0.015). **(c)** Wildtype behavior towards different tastants: larvae respond with aversion to 6 mM of caffeine, 10 mM of maltose and 25 mM of trehalose, but show no preference to arabinose (20 mM). **(d)** Ablation of B2 by

expression of proapototic genes *hid;rpr* does not change the observed avoidance to denatonium (5 mM) (*B2*-Gal4>+/*hid;rpr*>+ p=0.561 (NS); *B2*-Gal4>+/*B2*-Gal4>*hid;rpr* p=0.836 (NS); *hid;rpr*>+/*B2*-Gal4>*hid;rpr* p=0.852 (NS)). Error bars show the SEM and significances are indicated as following: *** p<0.001, ** p<0.01, * p<0.05 in Wilcox signed rank test.

Supplementary table 1: Measurements with water and p-Values for all neurons (Calcium Imaging)

C7	
H2O	df/f (%)
date	
9.9.15	4.94
9.9.15.2	-0.94
9.9.15.3	7.35
9.9.15.4	-12.19
9.9.15.5	5.53
14.9.15	-9.27
Average:	-0.763333333

B2	
H2O	df/f (%)
date	new
29.9.15.3	-4.95
30.9.15	-0.12
30.9.15.2	2.69
30.9.15.3	-5.43
30.9.15.4	1.84
	-1.194

C2	
H2O	df/f (%)
date	
16.9.15	0.78
16.9.15.2	5.5
16.9.15.3	-17.75
6.10.15	3.7
6.10.15.2	0.36
Average:	-1.482

C3	
H2O	df/f (%)
date	
29.9.15	10.38
29.9.15.2	7.34
29.9.15.3	-2.6
30.9.15	-4.24
30.9.15.2	-1.17
30.9.15.3	-4.99
Average:	0.786666667

C6	
H20	df/f (%)
Date	
18.9.15	-1.93
18.9.15.2	-1.6
29.9.15	12.38
29.9.15.3	-5.86

29.9.15.2	3.03

Average: 1.204

Wilcoxon rank sum test

all data compared to H_2O only

C7			B2		
Substance	p value		Substance	p value	
KCl 1M	0.004329	**	KCl 1M	0.8413	
NaCl 10 mM	0.4452		NaCl 10 mM	0.6905	
NaCl 100 mM	0.09307		NaCl 100 mM	0.6905	
NaCl 1 M	0.002165	**	NaCl 1 M	0.6389	
Fructose 500 mM	0.8182		Fructose 500 mM	0.9271	
Glucose 500 mM	0.3142		Glucose 500 mM	0.1508	
Sucrose 500 mM	0.0003996	***	Sucrose 500 mM	0.3095	
Trehalose 25 mM	0.7922		Trehalose 25 mM	1	
Arabinose 20 mM	0.8182		Arabinose 20 mM	0.4206	
Maltose 10 mM	0.4848		Maltose 10 mM	0.1508	
Sorbitol 100 mM	0.4286		Sorbitol 100 mM	1	
Glycerol 10 %	0.004329	**	Glycerol 10 %	0.2222	
Quinine 5 mM	0.002165	**	Quinine 5 mM	0.007937	**
Caffeine 6 mM	1		Caffeine 6 mM	1	
Denatonium 10 mM	0.000666	***	Denatonium 10 mM	0.007937	**
Coumarine 10 mM	0.9307		Coumarine 10 mM	0.8413	
Canavanine 30 mM	0.08225		Canavanine 30 mM	0.1429	
Arginine 100 mM	0.329		Arginine 100 mM	0.2222	
Glutamate 100 mM	0.004329	**	Glutamate 100 mM	0.1161	
Leucine 100 mM	0.4286		Leucine 100 mM	0.1508	
Lysine 100 mM	0.004329	**	Lysine 100 mM	0.03175	*
Threonine 100 mM	1		Threonine 100 mM	0.6905	
Valine 100 mM	0.5368		Valine 100 mM	0.05556	
Citric acid 100 mM	0.000666	***	Citric acid 100 mM	0.007937	**
HCl 3.7 %	0.004329	**	HCl 3.7 %	1	

C2			C3		
Substance	p value		Substance	p value	
KCl 1M	0.004329	**	KCl 1M	0.4206	
NaCl 10 mM	0.5368		NaCl 10 mM	0.8413	
NaCl 100 mM	0.004998	**	NaCl 100 mM	0.5476	
NaCl 1 M	0.004329	**	NaCl 1 M	0.8413	
Fructose 500 mM	0.4286		Fructose 500 mM	1	
Glucose 500 mM	0.9307		Glucose 500 mM	0.8413	

Sucrose 500 mM	0.004329	**	Sucrose 500 mM	0.5476	
Trehalose 25 mM	0.6623		Trehalose 25 mM	1	
Arabinose 20 mM	0.7922		Arabinose 20 mM	1	
Maltose 10 mM	0.8182		Maltose 10 mM	0.3095	
Sorbitol 100 mM	0.6623		Sorbitol 100 mM	0.8413	
Glycerol 10 %	0.2468		Glycerol 10 %	0.5476	
Quinine 5 mM	0.9307		Quinine 5 mM	0.09524	
Caffeine 6 mM	0.329		Caffeine 6 mM	0.3095	
Denatonium 10 mM	0.7922		Denatonium 10 mM	0.4286	
Coumarine 10 mM	0.6623		Coumarine 10 mM	0.5476	
Canavanine 30 mM	0.5476		Canavanine 30 mM	0.7857	
Arginine 100 mM	0.002664	**	Arginine 100 mM	0.4206	
Glutamate 100 mM	0.6623		Glutamate 100 mM	0.8413	
Leucine 100 mM	0.004329	**	Leucine 100 mM	0.8413	
Lysine 100 mM	0.7308		Lysine 100 mM	1	
Threonine 100 mM	0.4286		Threonine 100 mM	0.9307	
Valine 100 mM	0.004329	**	Valine 100 mM	0.6905	
Citric acid 100 mM	0.5368		Citric acid 100 mM	0.6905	
HCl 3.7 %	0.7922		HCl 3.7 %	0.4206	

C6		
Substance	p value	
KCl 1M	0.007937	**
NaCl 10 mM	0.3095	
NaCl 100 mM	1	
NaCl 1 M	0.007937	**
Fructose 500 mM	1	
Glucose 500 mM	0.2222	
Sucrose 500 mM	0.01587	*
Trehalose 25 mM	0.329	
Arabinose 20 mM	0.4206	
Maltose 10 mM	0.6905	
Sorbitol 100 mM	0.4206	
Glycerol 10 %	0.004329	**
Quinine 5 mM	0.6905	
Caffeine 6 mM	0.8413	
Denatonium 10 mM	1	
Coumarine 10 mM	0.8413	
Canavanine 30 mM	0.3929	
Arginine 100 mM	0.8413	
Glutamate 100 mM	0.05556	
Leucine 100 mM	0.4633	
Lysine 100 mM	0.8413	
Threonine 100 mM	0.6905	
Valine 100 mM	0.2222	

Citric acid 100 mM	0.4206	
HCl 3.7 %	0.007937	**

Supplementary table 2: pH and osmolarity for all used tastants

Substance	Concentration	Osmolarity (mOsm/Kg)	pH
KCl	1 M	1765	5.2
NaCl	10 mM	15	8.6
	100 mM	194	6
	500 mM	939	5.9
	1 M	1890	5.2
Fructose	500 mM	525	4.2
Glucose	500 mM	530	6.2
	1M	1134	8
Sucrose	100 mM	99	4.9
	200 mM	176	5.1
	500 mM	566	6.1
	1M	1219	8.2
Trehalose	25 mM	24	8
Arabinose	20 mM	18	8.5
	100 mM	107	4.4
	200 mM	208	5.2
	500 mM	513	3.5
	1 M	1096	3.3
Maltose	10 mM	8	6
Sorbitol	100 mM	103	6
Glycerol	10%	1728	7.4
Quinine	5 mM	3	6.8
Caffeine	6 mM	1	5
Denatonium	10 mM	6	9.3
Coumarine	10 mM	8	6.1
L-Canavanine	30 mM	36	8
L-Arginine	100 mM	100	10
L-Glutamate	100 mM	171	7
L-Leucine	100 mM	101	5.8
L-Lysine	100 mM	147	5.4
L-Threonine	100 mM	46	5.5
L-Valine	100 mM	103	5.8
Citric acid	100 mM	111	2
HCl	3.70%	2161	2
